# Supplementary material for: Profiles of Loneliness and Social Isolation in Physically Active and Inactive Older Adults in Rural England
Source: Int J Environ Res Public Health. 2021 Apr 9;18(8):3971. doi: 10.3390/ijerph18083971 (PMC8070246; doi:10.3390/ijerph18083971)
Supplement: Supplementary file 1 [file ijerph-18-03971-s001.pdf]

Supplementary Material

TOPIC: SOCIAL AND PHYSICAL ACTIVITIES ACROSS THE LIFECOURSE AND AT PRESENT.

Thank you for agreeing to take part in this follow-up study. During this interview I will ask you to look back at the activities you have taken part in across your lifetime, about what led you to do these activities, and how they may have influenced you in later years.

Then I will ask some questions about the types of social and physical activities that you do in your current everyday life, what leads you to do these, and how your past experiences may have influenced these.

Part 1. Activities over the life course

[Timing]
1. Thinking back across your lifetime, when were the times that you were particularly active, with regards to social activity or physical activity?

- Cue: Were there other times, in which you were also active, but differently active?

Interviewer: If the participant mentions several different times in his/her lifetime, ask each of the following for each specific time:

- a) What type of activities were you doing then?
- b) Who did you do these activities with?
- c) What motivated you to do these activities?
- d) Were there any obstacles you had to overcome? How did you overcome them?
- e) Were there obstacles you didn't overcome? Why couldn't you overcome them?
- f) Is there anything else you think is important to mention about this time in your life?

[Process & Context]
2. Have there been times in your lifetime when changes in your roles or your situation prompted you to decrease of increase your level of activity or the types of activities you engaged in?

- Cue: such as finding work, getting married, having children, moving away etc.

Interviewer: For each transition the participant mentions, ask each of the following questions:

- a) How did your activities change?
- b) What was or were the reasons that this transition influenced your activities?
- c) How did you find this transition?

- d) Were there any consequences to this change in activities? Physically? Socially? Emotionally?
- e) How do you think this transition has affected the activities you started, stopped or continued to do in later years?
- f) Is there anything else about this life transition which you think is important to mention?

Part 2. Relating current activities with the life course

Interviewer: Substitute each of the ______ with the 5 most frequent activities the participant noted down in the 7-day activity diary.

3. In the 7-day Activity Diary which you completed in the first study, you noted down that _______, _______, ______, ______ and ______ are activities which frequently take you out of the house in the week.

- a) Would you say that this is accurate for you as you are now?

Interviewer, skip (b) – (c) if participant agrees that this is correct.

- b) If not, could you tell me what other activities take you out of the house on a weekly basis?
- c) How many times per week does this activity take you out of the house?
- d) With whom do you do these activities?
- e) How do you travel to and from these activities?

Interviewer: Ask the next set of questions for each frequent activity, starting with the most frequent.

4. Now, I'd like to ask you some questions about each activity which takes you out of you house during a normal week.

Let's start with _______ (*e.g. Going to Church).*

- a) What is your motivation for _______?
- b) Have any of your activities experiences in life previously played a role in how you currently _______? How so? ***[Timing]***
- b) Have any transitions you've had, in the past or more recently, had an effect on how you _______? How so? ***[Process]***
- c) Looking back in time, as well as at your present situation, what are the combination of factors which lead you to _______? ***[Context]***
- d) Are there any other things about _______ which you think are important to mention?

5. Are there any social or physical activities which you did until recently, say within the last 20 years, which you have stopped doing regularly?

- a) When did you stop? ***[Timing]***
- b) When did you start doing this activity? ***[Timing]***
- c) At this time, what motivated you start and continue this activity? ***[Context]***
- d) What is the reason that you stopped taking part in this activity? ***[Process]***

Cue 1: Was it a change in role or life situation which brought on this change?
Cue 2: How so?

- e) Is there anything else which you would like to mention about this activity, or about your life situation when you stopped doing it?
